# Inhibitory Effect of a Hot-Water Extract of Leaves of Japanese Big-Leaf Magnolia (*Magnolia obovata*) on Rotavirus-Induced Diarrhea in Mouse Pups

**DOI:** 10.1155/2014/365831

**Published:** 2014-12-15

**Authors:** Takeshi Kawahara, Takuma Tomono, Yasunori Hamauzu, Katsumi Tanaka, Hisako Yasui

**Affiliations:** ^1^Department of Sciences of Functional Foods, Graduate School of Agriculture, Shinshu University, 8304 Minamiminowa, Kamiina, Nagano 399-4598, Japan; ^2^Department of Interdisciplinary Genome Sciences and Cell Metabolism, Institute for Biomedical Sciences, Interdisciplinary Cluster for Cutting Edge Research (IBS-ICCER), Shinshu University, 8304 Minamiminowa, Kamiina, Nagano 399-4598, Japan; ^3^Kiso Town Resources Institute, 2326-6 Fukushima Kisomachi, Kiso-gun, Nagano 397-8588, Japan

## Abstract

The leaf of Japanese big-leaf magnolia (*Magnolia obovata* Thunb.) has long been used as a natural packaging material for traditional foods in Japan. However, many of the physiological functions of the leaves against oral infection and resultant illness remain unclear. The aim of the present study was to investigate the effects of a hot-water extract of the leaves of *Magnolia obovata* on diarrhea induced by rotavirus (RV), a major cause of acute diarrhea. RV strain SA11 was mixed with the *M. obovata* leaf extract and inoculated orally to neonatal BALB/c mouse pups. Simultaneous inoculation of SA11 with the extract significantly decreased the incidence of diarrhea. In addition, the extract significantly inhibited cytopathic effects and mRNA expression of viral proteins in SA11-infected MA104 cells. Two flavonoid glycosides, quercitrin and rutin, were strongly suggested to be major anti-RV agents in the extract by serial solvent extraction and reversed-phase HPLC-ESI-MS analysis. Our results suggest that the hot-water extract of *M. obovata* leaves can be used as a medicine or food additive to prevent and ameliorate RV-induced diarrhea in individuals that may have difficulty in benefitting from the RV vaccines.

## 1. Introduction

Japanese big-leaf magnolia (*Magnolia obovata* Thunb.) is a deciduous shrub belonging to family Magnoliaceae. The dried bark of* M. obovata* is used as a basic ingredient of the crude herbal drug known as “Wakoboku” in Japan [1, 2]. In addition, the leaf of* M. obovata* called “Hooba” is used as a natural packaging material for traditional foods such as “Hoobamaki” and “Hooba Miso.” Given their usage as food packaging, the leaves of* M. obovata* are potentially effective to prevent illness caused by oral infection. However, many of the physiological functions of* M. obovata* leaves against oral infection and resultant illness remain unclear.

Rotavirus (RV), a double-stranded RNA virus in the family Reoviridae, is one of the most frequent causes of acute diarrhea in infants and children worldwide [3]. According to the World Health Organization, RV is responsible for more than 600,000 diarrhea-related childhood deaths each year [4]. Transmission of RV occurs through fecal-oral contact with contaminated stool [5]. Once RV infection has occurred, it is difficult to prevent transmission, because this virus is stable in feces and relatively resistant to common disinfectants [6]. In order to prevent RV infection, 2 RV vaccines, Rotarix and RotaTeq, are currently approved in many countries [7, 8]. However, these vaccines are still expensive to introduce into developing countries [9], and the immunization schedule of these vaccines must be strictly controlled to avoid intussusception. Therefore, many researches have been undertaken to search for new effective routinely available and inexpensive anti-RV compounds from herbal products [10–12].

In the present study, we investigated the effect of a hot-water extract prepared from* M. obovata* leaves on RV-induced diarrhea in neonatal BALB/c mouse pups. Furthermore, we tested the ability of MLE to suppress cytopathic effects (CPE) and amplification of viral protein mRNA in SA11-infected MA104 cells.

## 2. Methods

### 2.1. Preparation of* M. obovata* Leaf Extract (MLE)

The leaves of* M. obovata* were harvested in the Kiso area of Nagano, Japan. After removal of leaf stems, the leaves were cut into pieces, frozen in liquid nitrogen, and ground into a fine powder in a mortar. The powdered leaves were suspended in 10 times their volume of water and extracted at 100°C for 1 h. The extract was centrifuged at 5000 g for 30 min and the supernatant was collected. The supernatant was filtered using a cellulose acetate membrane filter with a 0.2 *μ*m pore size (Advantec, Tokyo, Japan). The obtained MLE was lyophilized and stored under light-shielded conditions until use.

### 2.2. Cells and Viruses

Rhesus monkey kidney cell line MA104 was cultured in monolayer cultures using Eagle's minimal essential medium (MEM; Nissui Pharmaceutical, Tokyo, Japan) containing 10% heat-inactivated fetal bovine serum (FBS; Equitech-Bio, Kerrville, TX, USA) and 2 M l-glutamine. Cells were passaged using conventional procedures with 0.05% trypsin at 37°C in a humidified atmosphere (5% CO_2_/95% air).

Rhesus RV strain SA11 (group A, type III) was propagated in MA104 cells and harvested after 2 freeze-thaw cycles. Viral titer (TCID_50_/mL) was determined by neutral red uptake assay.

### 2.3. Mice

Specific pathogen-free pregnant BALB/c mice aged 6 weeks were purchased from Japan SLC (Shizuoka, Japan) and housed at 23°C ± 3°C under a 12 h light/dark cycle. Mice were housed one per cage prior to giving birth. All animal protocols were approved by the Committee for Animal Experiments of Shinshu University.

### 2.4. RV-Induced Diarrhea

The effects of MLE on RV-induced diarrhea were evaluated according to the previously described method [13]. Briefly, pregnant mice were separately housed and fed with the MM-3 diet (Funabashi Farm, Chiba) until they gave birth. Seven days after birth, parent mice and pups were divided into 2 groups that each consisted of 1 parent mouse and 7 pups. The pups were infected with RV by oral administration of 50 *μ*L of the SA11 strain suspension, with or without 50 *μ*L of 1 mg/mL MLE dissolved in DW, for 1 h. The incidence of SA11-induced diarrhea in pups was determined by checking stool consistency for 7 consecutive days. Stool consistency was evaluated on a 2-point scale (0: normal solid with black color; 1: loose or liquid with light brown color), and cumulative incidence rate was estimated. Mice demonstrating level 1 stools were considered positive for diarrhea.

### 2.5. Cytopathic Effect (CPE) Inhibition Assay

**Figure 1:**
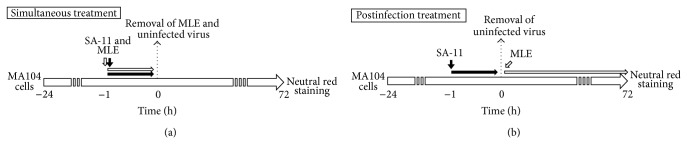
Protocol for MLE treatment in RV infection study using MA104 cells.

To investigate whether MLE have inhibitory effect on RV infection, CPE on MA104 cells produced by the infection of SA11 strain was evaluated by microscopic observation and neutral red uptake assay [14]. MA104 cells (7.2 × 10^4^ cells/200 *μ*L) were seeded onto 96-well tissue culture plates (BD Falcon, Franklin Lakes, NJ, USA) and cultured. After 48 h, the cells were treated with MLE and SA11 strain under simultaneous conditions or postinfection treatment conditions as shown in Figure 1.

The treated cells were incubated with 150 *μ*g/mL neutral red dye/Hank's balanced salt solution in dark for 2 h. After removing neutral red solution, cells were washed with 100 *μ*L of PBS. To release intracellular dye, destaining solution (1 : 1 solution of 1% acetic acid/50% ethanol) was added to the wells at room temperature for 30 min. The released dye was transferred to a 96-well assay plate (Thermo Fisher Scientific, Roskilde, Denmark) and absorbance at 570 nm was measured using an iMark microplate reader (Bio-Rad Laboratories, Hercules, CA, USA).

### 2.6. Characterization of the Anti-RV Component in MLE

**Figure 2:**
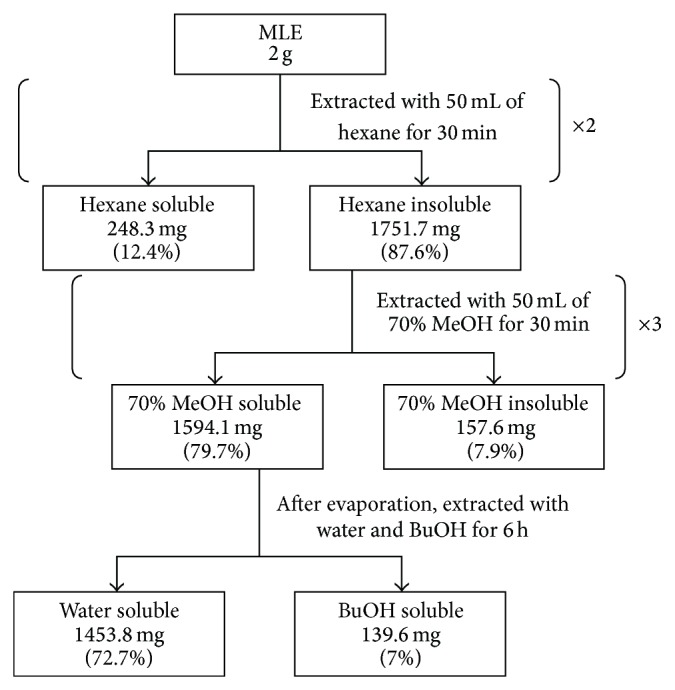
Fractionation protocol for the characterization of anti-RV agent in MLE.

To identify the anti-RV component in MLE, the extract was subjected to fractionation as shown in Figure 2. Individual fractions were concentrated under reduced pressure in a rotary evaporator N-1000 (EYELA, Tokyo, Japan).

### 2.7. Quantitative RT-PCR Assay

To examine the effects of MLE on the amplification of mRNA for RV constitutive protein VP6 and non-structural protein 4 (NSP4), MA104 cells (2.8 × 10^5^ cells/mL/well) treated with MLE and SA11 strain (1.0 × 10^2^ TCID_50_) in 24-well plates (BD Falcon) for indicated times were subjected to quantitative RT-PCR assay.

Total RNA was extracted from cells using TRIzol reagent (Life Technologies, Carlsbad, CA, USA) according to the manufacturer's protocol. The extracted RNA (1 *μ*g) was reverse transcribed in a thermal cycler (PTC-200; MJ Research, Waltham, MA, USA) with 1 mM each dNTP, 2.5 units/*μ*L M-MLV reverse transcriptase (Invitrogen), and 10 pmol/*μ*L of oligo(dT)18 primers at 42°C for 50 min.

Quantitative RT-PCR of the resulting cDNA was performed using 0.5 *μ*g of cDNA with a SYBR Premix Ex Taq (Takara Bio, Shiga, Japan) and 10 pmol/*μ*L primers. The primer sequences for Simian RV VP6 [GenBank: L15384.1] were designed as 5′-CTTCTACCAGACGCGGAAAG-3′ (forward) and 5′-ATTCGGCCTGAGAATCACTG-3′ (reverse) and were complementary to 696–715 and 794–775, respectively. The primer sequences for Simian RV NSP4 [GenBank: AF087678.1] were designed as 5′-ATAAATTGACGGTGCAAACG-3′ (forward) and 5′-TGCTGCAGTCACTTCTCTTG-3′ (reverse) and were complementary to 395–414 and 522–503, respectively. The primer sequences for* Macaca mulatta* glyceraldehyde-3-phosphate dehydrogenase [GAPDH; GenBank: NM_001195426.1] were designed as 5′-TCAACGACCACTTTGTCAAG-3′ (forward) and 5′-GCCAAATTCGTTGTCATACC-3′ (reverse) and were complementary to 968–987 and 998–1017, respectively. The PCR comprised 1 cycle of preheating (95°C, 10 min) and 40 cycles of denaturation (95°C, 10 s), primer annealing, and extension (55°C, 30 s) using an Eco Real Time PCR System (Illumina, San Diego, CA, USA). Results were analyzed with the ΔΔCt method using Eco system software (Illumina). The amounts of PCR products were normalized to the expression level of the GAPDH gene.

### 2.8. Estimation of the Amount of Total Phenolics

The amount of total phenolics in MLE and fractionated MLE was determined by the Folin-Ciocalteu method. Briefly, 150 *μ*L of each sample (0.1 mg/mL) was mixed with 150 *μ*L of Folin-Ciocalteu reagent and left for 3 min at room temperature. Next, 150 *μ*L of 10% aqueous sodium bicarbonate solution was added to the mixture, which was left for 1 h at room temperature. The absorbance of the mixture was measured at 700 nm using an Ultrospec 3300 pro UV/Visible Spectrophotometer (Amersham Pharmacia Biotec, Uppsala, Sweden). Data were expressed as gallic acid equivalent.

### 2.9. Reversed-Phase HPLC

The HPLC analyses were conducted using a liquid chromatography system (Shimadzu, Kyoto, Japan) equipped with a PDA detector (SPD-M10Avp photodiode array detector). Results were acquired and processed using Shimadzu Workstation CLASS-VP v.1.0 software (Shimadzu). Chromatographic separation was carried out on a Luna 5 *μ* C18 column (150 × 4.6 mm, Phenomenex, Torrance, CA, USA) with a security guard cartridge (3.0 × 4.6 mm) at 40°C. Solvents were 0.1% trifluoroacetic acid (A) and 0.1% trifluoroacetic acid in acetonitrile (B). The gradient program began with 0% B and was changed to obtain 8% B at 10 min, 10% B at 30 min, 25% B at 45 min, 75% B at 50 min, and 90% B at 65 min. The flow rate was 1.0 mL/min and the injection volume was 20 *μ*L. Detection was performed at 280 nm, 325 nm, 354 nm, and 370 nm. Peaks were identified by comparing retention times and UV-visible spectra with those of standards. The HPLC-electrospray ionization mass spectrometry (HPLC-ESI-MS) system was also used for peak identification.

### 2.10. LC-ESI-MS

LC-ESI-MS analysis was performed on an ACQUITY UPLC separation module coupled with a Micromass Quattro Micro API mass spectrometer (Waters, Milford, MA, USA). The column was a Luna 5 *μ* C18 (150 × 4.6 mm) with a security guard cartridge (3.0 × 4.6 mm) and the mobile phases consisted of 0.1% formic acid (solvent A) and acetonitrile containing 0.1% formic acid (solvent B). The flow rate was 0.3 mL/min. The gradient method was the same as previously described for HPLC-PDA analysis. ESI-MS detection was performed under negative ion mode under the following conditions: capillary voltage: −3 kV; cone voltage: −50 V; source temperature: 120°C; desolvation temperature: 350°C; cone gas flow: 50 L/h; desolvation gas flow: 600 L/h. MS data collected were processed using MassLynx v.4.1 software (Waters).

### 2.11. Statistical Analysis

The incidence of diarrhea was analyzed using Fisher's exact test. The antiviral activity data were statistically analyzed using two-tailed Student's *t*-test. *P* values less than 0.05 were considered to be statistically significant.

## 3. Results

### 3.1. Effect of MLE on the Incidence of RV-Induced Diarrhea

**Figure 3:**
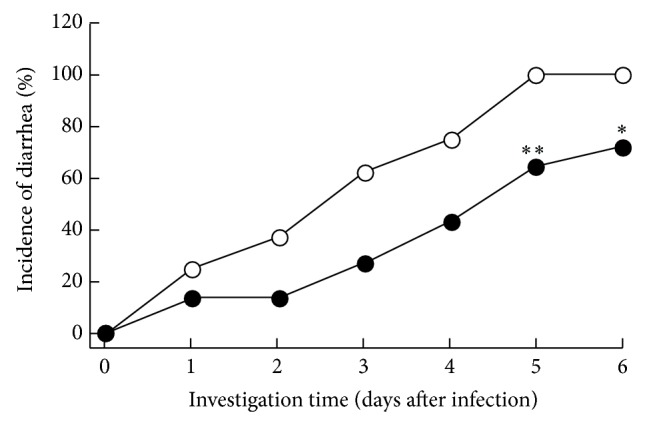
Effect of MLE on the incidence of RV-induced diarrhea. RV strain SA11 was mixed with the MLE or water (control) and inoculated orally to neonatal BALB/c mouse pups. The incidence of diarrhea of control mice was evaluated for 7 consecutive days. Results obtained from control group and MLE-treated group were represented by white circles and black circles, respectively. Data are presented as mean ± SD (*n* = 7 per group). ^*^ *P* < 0.05 versus control group.

We obtained MLE as a dried powder from the powdered* M. obovata* leaves with a 7.8% yield on a wet basis. As shown in Figure 3, mouse pups inoculated with the SA11 strain started to develop diarrhea 1 day after the inoculation, which persisted to the termination of the experiment. In the control group, all mice developed diarrhea 5 days after inoculation. In contrast, 71.8% of mice inoculated with MLE showed diarrhea. Five to six days after inoculation, the incidence of diarrhea in the MLE-treated group was significantly (*P* < 0.05) lower than that in the control group.

### 3.2. Effect of MLE on CPE in SA11-Infected MA104 Cells

**Figure 4:**
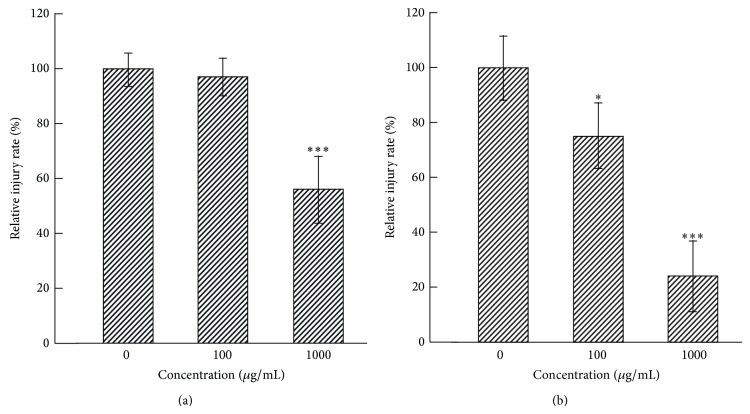
Effect of MLE on CPE in SA11-infected MA104 cells. MA104 cells were treated with MLE simultaneously or 1 h after the exposure to SA11 strain. The cells were cultured for another 72 h and subjected to neutral red uptake assay to measure CPE produced by SA11 infection. (a) Simultaneous treatment. Data are presented as relative mean value ± SD against MLE-untreated control (*n* = 3). ^***^ *P* < 0.001 versus MLE-untreated control. (b) Postinfection treatment. Data are presented as relative mean value ± SD against MLE-untreated control (*n* = 3). ^*^ *P* < 0.05 and ^***^ *P* < 0.001 versus MLE-untreated control.

We used MLE up to a concentration of 1000 *μ*g/mL for investigations because the 50% cytotoxic concentration of MLE on MA104 cell growth was calculated to be 10.3 mg/mL (data not shown). In the CPE inhibition assay, 100 and 1000 *μ*g/mL of MLE inhibited CPE by approximately 7% and 44%, respectively, under simultaneous treatment conditions (Figure 4(a)). The inhibition produced by 1000 *μ*g/mL MLE was statistically significant compared to that by MLE-untreated control. Postinfection treatment with 100 and 1000 *μ*g/mL of MLE significantly inhibited CPE by 25% and 76%, respectively (Figure 4(b)). The 50% inhibitory concentration (IC_50_) for MLE inhibition of CPE was calculated to be 1142 *μ*g/mL for the simultaneous treatment and 575 *μ*g/mL for the postinfection treatment.

### 3.3. Effect of Fractionated MLE on CPE in SA11-Infected MA104 Cells

**Figure 5:**
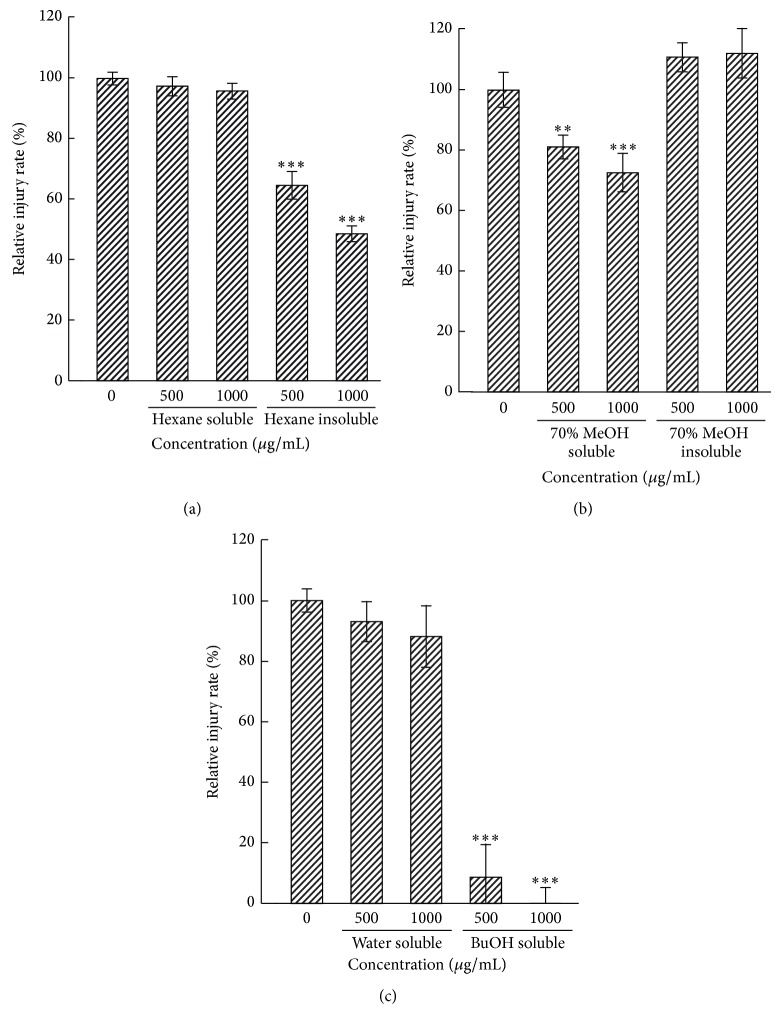
Effect of fractionated MLE on CPE in SA11-infected MA104 cells. MA104 cells were treated with fractionated MLE 1 h after the exposure to SA11 strain. The cells were cultured for another 72 h and subjected to neutral red uptake assay to measure CPE produced by SA11 infection. (a) Effects of hexane-soluble and hexane-insoluble MLE fraction on cytopathic injury. Data are presented as relative mean value ± SD against fraction-untreated control (*n* = 3). ^***^ *P* < 0.001 versus fraction-untreated control. (b) Effects of the 70% MeOH-soluble and 70% MeOH-insoluble fractions on cytopathic injury. Data are presented as relative mean value ± SD against the fraction-untreated control (*n* = 3). ^**^ *P* < 0.01 and ^***^ *P* < 0.001 versus fraction-untreated control. (c) Effects of water-soluble and 1-BuOH-soluble MLE fractions on cytopathic injury. Data are presented as relative mean value ± SD against the fraction-untreated control (*n* = 3). ^***^ *P* < 0.001 versus fraction-untreated control.

The yields of the fractionated MLE are shown in Figure 2. After hexane extraction, the insoluble fraction at concentrations of 500 and 1000 *μ*g/mL dose-dependently inhibited CPE in MA104 cells by 35% and 52%, respectively. In contrast, the hexane-soluble fraction had a negligible effect on CPE at concentrations up to 1000 *μ*g/mL (Figure 5(a)). The 70% MeOH-soluble fraction obtained from the hexane-insoluble fraction inhibited CPE by 11% and 24% at concentrations of 500 and 1000 *μ*g/mL, respectively (Figure 5(b)). In contrast, the 70% MeOH-insoluble fraction did not inhibit CPE. After liquid-liquid extraction of the 70% MeOH-soluble fraction using water and 1-BuOH, the 1-BuOH-soluble fraction significantly inhibited CPE (Figure 5(c)). CPE inhibition rates of approximately 93% and 100% were produced by concentrations of 500 and 1000 *μ*g/mL, respectively, of the 1-BuOH-soluble fraction, and these reductions were statistically significant. The other water-soluble fraction did not affect CPE.

### 3.4. Effect of 1-BuOH-Soluble Fraction of MLE on the Amplification of Viral Protein mRNA in SA11-Infected MA104 Cells

**Figure 6:**
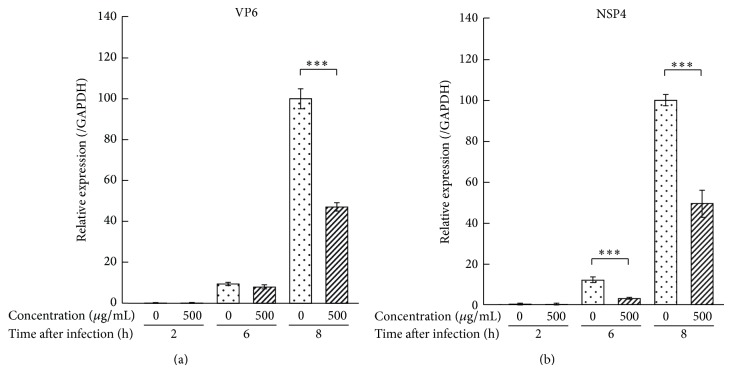
Effect of 1-BuOH-soluble fraction of MLE on the amplification of viral protein mRNA in SA11-infected MA104 cells. MA104 cells were treated with 1-BuOH-soluble fraction of MLE 1 h after the exposure to SA11 strain. The SA11-infected cells were cultured for other indicated times and subjected to quantitative RT-PCR assay to evaluate the expression of VP6 and NSP4. Data are presented as relative mean value ± SD against fraction-untreated control after 8 h (*n* = 3). ^***^ *P* < 0.001 versus 1-BuOH-soluble fraction-untreated counterpart.

As shown in Figure 6, 53% of VP6 expression after 8 h of SA11 infection was inhibited by treatment with 500 *μ*g/mL of the 1-BuOH-soluble fraction, whereas the expression was not affected within 6 h after infection. The expression of NSP4 was decreased by 49% by treatment with the 1-BuOH-soluble fraction for 6 h or more.

### 3.5. HPLC Profiles of the 1-BuOH-Soluble Fraction of MLE

The phenolic content per 1 mg of the MLE, the water-soluble fraction, and the 1-BuOH-soluble fraction were estimated at 49.6, 9.5, and 172.5 *μ*g gallic acid equivalent, respectively. To further characterize the results from the Folin-Ciocalteau assay, the 1-BuOH-soluble fraction was subjected to RP-HPLC analysis to detect phenolic compounds.

**Figure 7:**
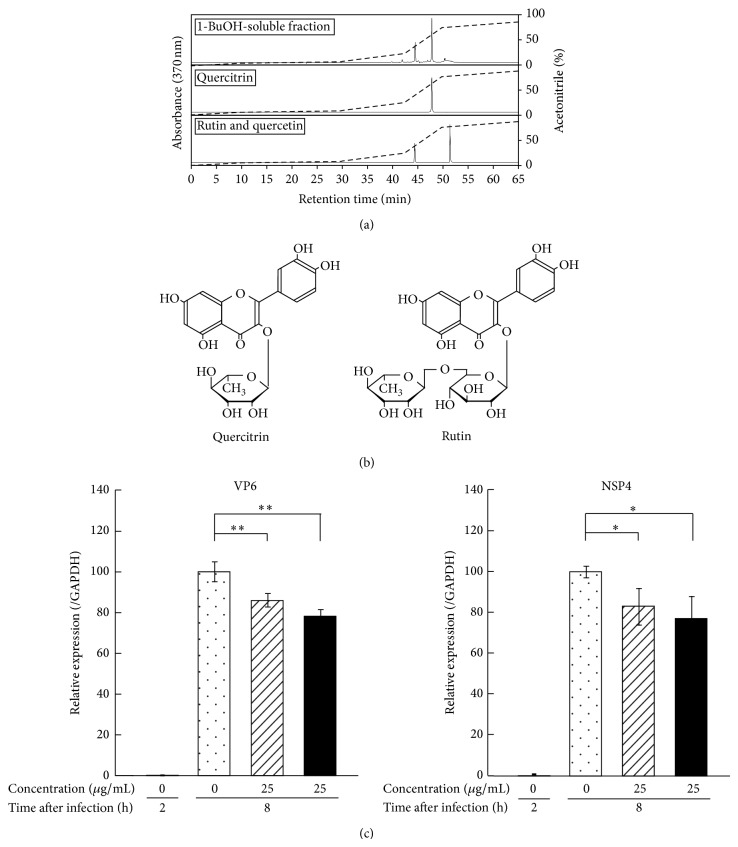
Anti-RV agents in 1-BuOH-soluble fraction of MLE. (a) HPLC profiles of 1-BuOH-soluble fraction of MLE, quercitrin, and rutin and quercetin. The* x*-axis represents time and the* y*-axis represents voltage. (b) Structures of quercitrin and rutin. (c) Effect of quercitrin and rutin on the amplification of viral protein mRNA in SA11-infected MA104 cells. MA104 cells were treated with quercitrin and rutin 1 h after the exposure to SA11 strain. The SA11-infected cells were cultured for another 8 h and subjected to quantitative RT-PCR assay to evaluate mRNA expression of VP6 and NSP4. The results of untreated (control) cells, quercitrin-treated cells, and rutin-treated cells were shown by dotted bar, shaded bar, and solid black bar, respectively. Data are presented as relative mean value ± SD against untreated control after 8 h (*n* = 3). ^*^ *P* < 0.05 and ^**^ *P* < 0.01 versus untreated counterpart.

HPLC analysis showed that the 1-BuOH fraction contained 2 flavonol derivatives (P1 and P2) as the main phenolics (Figure 7(a)). The retention time and absorption spectra of P1 and P2 were consistent with those of rutin and quercitrin. Moreover, from LC-MS analysis, a molecular ion peak [M-H]^−^ (at* m/z* 609 for P1 and at* m/z* 447 for P2) with a fragment ion peak observed at* m/z* 301 (corresponding to the deprotonated quercetin aglycone) revealed that P1 and P2 were rutin and quercitrin, respectively (Figure 7(b)). The concentrations of quercitrin and rutin in 500 *μ*g/mL MLE were estimated to be 15 *μ*g/mL and 7 *μ*g/mL, respectively.

### 3.6. Effect of Quercitrin and Rutin on the Expression of Viral Proteins in SA11-Infected MA104 Cells

To elucidate the roles of quercitrin and rutin in the anti-RV activity of the 1-BuOH-soluble fraction, the effects of these compounds at 25 *μ*g/mL on the expression of RV proteins were assessed. As shown in Figure 7(c), quercitrin and rutin significantly inhibited the amplification of VP6 and NSP4 expression in MA104 cells after 8 h of SA11 infection.

## 4. Discussion

In this study, we investigated the effect of MLE on the incidence of RV-induced diarrhea. The Simian RV strain SA11 was chosen because this strain is well characterized as a class A RV and is one of the most widely used reference strains in laboratories throughout the world [15]. Infection of target cells by the SA11 strain depends on sialic acids, which enables this strain to infect cells from a range of animal species [16]. Therefore, we used MA104 cells for infection assay. MA104 cells are widely used for the growth and characterization of animal culture-adapted RV strains [15].

The incidence of RV-induced diarrhea in SA11-infected mouse pups was reduced by administration of MLE. In addition, MLE and its 1-BuOH-soluble fraction suppressed the amplification of VP6 and NSP4 mRNA in SA11-infected MA104 cells, suggesting that the protective effect of MLE on RV-induced diarrhea is attributed to its broad suppressive effect on amplification of virus in the early stage of infection and not to specific suppression of biological function of enterotoxin NSP4 [17].

*M. obovata* bark has been reported to contain antiviral compounds honokiol and magnolol [18, 19]. However, these compounds were quite hydrophobic and insoluble in water [20]. In fact, our study strongly suggests that the anti-RV agents in MLE are quercitrin (quercetin 3-*O*-rhamnoside) and rutin (quercetin 3-*O*-rutinoside). These flavonoid glycosides have been reported as phenolic constituents in leaves, but not the bark, of* M. obovata* [21]. Therefore, the anti-RV effect of* M. obovata* leaves found in this study is suggested to be distinct from the known antiviral effect of its bark.

The relationship between the structure of flavonoids and their anti-RV effects has been investigated, and rutin has been reported to have anti-RV activity [22]. In this report, flavonoids with the* O*-rutinoside moiety, including rutin, showed inhibitory activity greater than that of their aglycones and were speculated to prevent the entry of RV and/or interaction of RV with target cells. Although quercitrin has not been reported to inhibit RV, it has been shown to inhibit other viruses [23–25]. In terms of antiviral mechanisms, quercitrin inhibited viral replication in the initial stage of infection by indirect interaction with virus particles [23]. Therefore, the anti-RV effects of MLE in spontaneous treatment and postinfection treatment shown in this study were attributed to the synergistic inhibitory effects of quercitrin and rutin.

Both quercitrin and rutin are glycosides formed from the flavonoid quercetin, and they were glycosylated at ring position 3. The common aglycone compound quercetin was reported to reduce infectivity and intracellular replication of RV [26]. However, quercetin needs the protection of a hydroxyl group at ring position 3 to continuously exert antiviral effects in aqueous solutions, because it undergoes oxidative degradation [27]. Therefore, the glycosylation of quercitrin and rutin at ring position 3 is considered to be important for maintenance of their anti-RV effects under aqueous conditions.

Flavonoid glycosides with* O*-rhamnoside and* O*-rutinoside moieties have low bioavailability because they are not hydrolyzed by endogenous enzymes [28] and are thus thought to pass through the small intestine and to be absorbed in colon as the aglycone quercetin or low-molecular weight phenolic acids after hydrolyzation by microflora [29, 30]. These properties of quercitrin and rutin may contribute to their protective effects on epithelial cells in the small intestine, which is the major target for RV infection, without degradation, as described previously [23]. The protective effect of MLE on RV-induced diarrhea* in vivo* can also be explained by the prompt suppressive effect of quercitrin and rutin on viral replication in target cells.

## 5. Conclusion

We found the anti-RV activity of* M. obovata* leaves. MLE effectively prevented RV infection to target cells and reduced the incidence of RV-induced diarrhea. We believe that* M. obovata* leaves represent effective starting materials for the development of anti-RV medicines or food additives to prevent RV-induced diarrhea in individuals that may have difficulty in benefitting from the RV vaccines.

## Abbreviations

CPE: Cytopathic effects
FBS: Fetal bovine serum
GAPDH: Glyceraldehyde-3-phosphate dehydrogenase
MLE: *Magnolia obovata* leaf extract
NSP4: Nonstructural protein 4
RV: Rotavirus
VP6: Viral protein 6.
